# Computational analysis of candidate prion-like proteins in bacteria and their role

**DOI:** 10.3389/fmicb.2015.01123

**Published:** 2015-10-15

**Authors:** Valentin Iglesias, Natalia S. de Groot, Salvador Ventura

**Affiliations:** Departament de Bioquìmica i Biologia Molecular, Institut de Biotecnologia i Biomedicina, Universitat Autònoma de BarcelonaBarcelona, Spain

**Keywords:** prion, bacteria, protein aggregation, pathogenesis, amyloid

## Abstract

Prion proteins were initially associated with diseases such as Creutzfeldt Jakob and transmissible spongiform encephalopathies. However, deeper research revealed them as versatile tools, exploited by the cells to execute fascinating functions, acting as epigenetic elements or building membrane free compartments in eukaryotes. One of the most intriguing properties of prion proteins is their ability to propagate a conformational assembly, even across species. In this context, it has been observed that bacterial amyloids can trigger the formation of protein aggregates by interacting with host proteins. As our life is closely linked to bacteria, either through a parasitic or symbiotic relationship, prion-like proteins produced by bacterial cells might play a role in this association. Bioinformatics is helping us to understand the factors that determine conformational conversion and infectivity in prion-like proteins. We have used PrionScan to detect prion domains in 839 different bacteria proteomes, detecting 2200 putative prions in these organisms. We studied this set of proteins in order to try to understand their functional role and structural properties. Our results suggest that these bacterial polypeptides are associated to peripheral rearrangement, macromolecular assembly, cell adaptability, and invasion. Overall, these data could reveal new threats and therapeutic targets associated to infectious diseases.

## Introduction

An increasing number of human diseases are being associated with amyloid forming proteins. Despite these polypeptides are diverse in function, sequence and origin, all share the propensity to form β-sheet aggregates (Karran et al., 2011). Amyloid fibril forming proteins appear to be highly conserved and have been detected in all kingdoms of life, suggesting that, despite they are usually thought to be involved in pathogenic processes, they might indeed provide selective advantages (Sanchez de Groot et al., 2012, 2015; Espinosa Angarica et al., 2013; Malinovska et al., 2013). In fact, cells exploit the formation of amyloid fibrils for diverse purposes (Coustou et al., 1997; Iconomidou et al., 2000; Podrabsky et al., 2001; Chapman et al., 2002; Graether et al., 2003; Fowler et al., 2006; Maji et al., 2009), from structure scaffolding, such as the melanin at the skin, to heritable information transmission, such as the yeast prions (Chien and Weissman, 2001; Shorter and Lindquist, 2005; Liebman and Chernoff, 2012; Staniforth and Tuite, 2012). Because amyloid fibers and their unstable intermediates can be highly cytotoxic (e.g., by disrupting the membrane integrity), the assembly of functional amyloids is a process tightly regulated by the cell, which involves the assistance of chaperones and a spatiotemporal control (Blanco et al., 2012; Gsponer and Babu, 2012; Evans et al., 2015; Taylor and Matthews, 2015).

Among amyloids, prions are a singular subset of proteins able to change from one conformational state to another, often an amyloid aggregate, and transmit it to other homologous polypeptide sequences. Importantly, recent results suggest that amyloid proteins involved in Alzheimer's and Parkinson's diseases could be infectious and act as prion-like proteins in the brain (Chiti and Dobson, 2006; Stöhr et al., 2012). With the exception of the mammalian prion protein (PrP), prion-like proteins constitute a subset of aggregation-prone proteins with special sequential composition. Whereas, classical amyloid proteins contain specific regions rich in hydrophobic residues that lead the protein self-assembly, prion-like proteins exhibit domains that are commonly enriched in asparagine and glutamine (Q/N) (Dorsman et al., 2002; Fändrich and Dobson, 2002; Halfmann et al., 2011) but also in glycine, serine and tyrosine residues (Kato et al., 2012). This pattern has been found in human proteins associated to neurodegenerative diseases, such as FUS (dementia) or TDP43 (amyotrophic lateral sclerosis; Kato et al., 2012). This special residue content results in low complexity sequences displaying disordered structures, a crucial property that ensures conformational flexibility, permits self-assembly without a requirement for conformational unfolding and allows conversion between species (Tompa and Fuxreiter, 2008; Fuxreiter, 2012; Fuxreiter and Tompa, 2012; Malinovska et al., 2013). In fact, one of the main evolutionary strategies to control protein aggregation is to ensure a stable globular structure preventing, in this way, the exposition of aggregation prone stretches (Lim and Sauer, 1991; de Groot and Ventura, 2005; Ventura, 2005; Monsellier et al., 2007). However, a polypeptide sequence requires more than just low complexity to behave as a prion (Espinosa Angarica et al., 2013; Malinovska et al., 2013). Hence, it has been found that the propagation of amyloid aggregation depends on characteristics such as the degree of over/under representation of specific residues and the length of the considered low complexity region (Ross et al., 2004, 2005; Toombs et al., 2010).

The scientific community is getting closer to elucidate the characteristics that differentiate prion and non-prion amyloid proteins (Kushnirov et al., 2007; Newby and Lindquist, 2013; Sabate et al., 2015). In this way, the knowledge acquired in the last 5 years has allowed the design of prediction approaches to identify putative prion proteins. The first predictive algorithms were based on the properties of the primary sequence responsible for the formation of the classical amyloid aggregates (e.g., high hydrophobicity and intrinsic β-sheet propensity). However, they failed to detect Q/N-rich stretches since these are polar residues that do not fulfill the typical requirements associated with classical β-sheet-amyloid aggregation (Pawar et al., 2005). Then, the algorithms focused on localizing Q/N rich segments in the primary sequence (Michelitsch and Weissman, 2000; Harrison and Gerstein, 2003), without paying much attention to the contribution of the rest of residues (Ross et al., 2005), being unable to score the proteins in terms of their relative prionogenicity. A big improvement was achieved by combining computational approaches with the experimental validation of new proteins displaying *in vitro* prionic properties. This strategy enlarged the set of prionic sequences and permitted the refinement of the available theoretical models. Alberti and co-workers employed a hidden Markov model (HMM), based on the four *bona fide* yeast prions identified to that moment, obtaining 200 yeast protein candidates carrying putative prion domains (PrDs; Alberti et al., 2009). The *in vivo* and *in vitro* analysis of the top 100 candidates rendered 29 proteins that proved heritable switch and significant *in vivo* amyloid formation. We have recently exploited this experimentally curated dataset to develop a probabilistic model of PrDs able to discover prionogenic proteins in complete proteomes (Espinosa Angarica et al., 2013). We have implemented this model in a web-based algorithm called PrionScan able to handle with large sequence databases and predict prion-like sequence stretches in the proteomes annotated in UniprotKB (Espinosa Angarica et al., 2014). In a previous work, we employed this predictor to analyze all the proteomes reported until that moment (1536 organisms; Espinosa Angarica et al., 2014). We discovered 20540 new putative prions present in 10 different taxonomic divisions, supporting prions universality. We also observed that in most cases the ratio of proteins with prion-forming domains is less than 1% of the whole proteome. Thus, in Archaea and Viruses the number is less than 10 per proteome, while in Bacteria, Fungi, Plantae, and Animalia the range is from few tens to few hundreds, depending on the organisms. Interestingly, we observed that, in different organisms, the predicted PrDs are associated with different cellular components and biological processes supporting prionic properties being employed for diverse biological purposes.

Bacteria are ubiquitous in the world, adapted to multiple environments and able to growth in the most extreme conditions. Moreover, bacterial infection remains a leading cause of death in both Western and developing world (WorldHealthOrganisation, WHO)^1^. Understanding which bacteria proteins display prionic properties could help to understand bacterial biology and pathogenesis. Indeed, despite no genuine prion has been characterized so far for prokaryotes, it is clear that at least *E. coli* can generate infectious conformations of heterologous fungal prions (Sabaté et al., 2009; Garrity et al., 2010; Espargaro et al., 2012; Yuan et al., 2014). In an analogous manner, the formation of amyloids was initially thought to be restricted to eukaryotic cells, but after the first report demonstrating that the curli fibers that emerge from the surfaces of *E. coli* cells had the same physical properties as human amyloids (Chapman et al., 2002), the number of discovered bacterial proteins displaying this ability is steadily increasing (Otzen and Nielsen, 2008; Blanco et al., 2012; Schwartz and Boles, 2013). Moreover, it has been observed that bacterial amyloids can initiate the formation of amyloid aggregates upon interaction with diverse host proteins (Otzen and Nielsen, 2008; Hufnagel et al., 2013; Friedland, 2015; Hill and Lukiw, 2015). With the aim to understand better the potential relevance of bacterial PrDs, here we focus on study the 2200 putative prion proteins predicted by PrionScan within the taxon domain bacteria, as derived from the study of 839 bacterial proteomes. Specifically, we analyze the functions and structures associated to these proteins and discuss the possible advantages that they could provide, ensuring their evolutionary conservation.

## Material and methods

### Sequence dataset

Our database was comprised of Uniprot Knowledgebase (UniProt, 2015) entries included both in Swissprot and TrEMBL (update 2012_03) under the taxon domain bacteria in order to track the prion like domains present in bacterial proteomes.

### Discovering putative prion-like domains

PrionScan, an algorithm developed by our group and described previously (Espinosa Angarica et al., 2014), was used in order to predict prion-like domains. Employing a cutoff of 50 bits, we identified 2200 PrD (Table S1). Further analysis was made *a posteriori* in order to identify common traits including the Gene Ontology GO terms for the molecular functions, biological processes, and cellular components and relevant domains according to Pfam database. Pfam domains and GO terms were manually annotated and counted in the 2200 positive PrD containing bacterial proteins according to the Uniprot annotations (UniProt, 2015). Due to the large amount of individual Pfam domains, only those ones represented more than five times were considered in the analysis. Then, we grouped the domains by similarity in their cellular function or process. The list of 18 selected pathogenic bacteria was manually annotated by looking for evidences of a human pathogenic association at the NCBI (Table S2). Then we calculated the enriched characteristics of the PrD containing proteins associated to these bacteria. The enrichment of the different GO terms and domains was calculated as explained below. The enrichment values obtained with the proteins detected in the subset of pathogenic bacteria were compared with those obtained for the complete 2200 PrD containing proteins dataset.

### Statistics

The enrichment analysis was performed with GOStat (Beissbarth and Speed, 2004) against the goa_uniprot database (UniProt, 2015). Out of 2200 initial proteins, 244 (11.09%) were annotated. A *p*-value of 0.1 was set as a cut-off and a false discovery rate (Benjamini) test was performed to obtain it. The initial clustering was performed by classifying the obtained Gene Ontologies according to their category: biological process, cellular component or molecular functions. We calculated the enrichment factors (*EF*) for every GO term to show how much higher is the proportion of hits in relation to the background sample (the total number of proteins). Accordingly, the EF is the number of hits among PrDs (*n*^*l*^) divided by the number of annotated proteins in our list (*p*^*l*^) and subsequently divided by the ratio between the hits of that GO term in goa_annotation (*n*^*b*^) and the total number of proteins (*p*^*b*^) in this specific GO term:

$$\begin{array}{l} EF=\frac{\frac{n^{l}}{p^{l}}}{\frac{n^{b}}{p^{b}}}=\frac{n^{l}p^{b}}{n^{b}p^{l}} \end{array}$$

Only those GO terms with a log2-fold enrichment >0.5 were considered to be significant for their subsequent analysis.

## Results

### Identifying PrD in bacteria proteomes

We have analyzed 839 Bacteria proteomes containing a total of 860337 proteins with PrionScan, from which we detected 2200 putative prion proteins scoring higher than 50 bits in the algorithm scale (Espinosa Angarica et al., 2013) accounting for a 0.3% of the complete protein dataset. Interestingly, in the 18 selected pathogenic bacteria (Table S2) proteins containing PrDs are significantly more abundant (2.4%) and indeed they constitute 40% of all the detected PrDs (891 PrDs). Moreover, some specific pathogenic organisms appear to be specially enriched in PrDs: *Staphylococcus aureus* (18%), *Enterococcus faecalis (*10%), *Enterococcus faecium* (5%), or *Staphylococcus epidermidis* (3%). These data show the diversity of putative PrDs distribution and suggest certain associated functionality.

**Figure 1:**
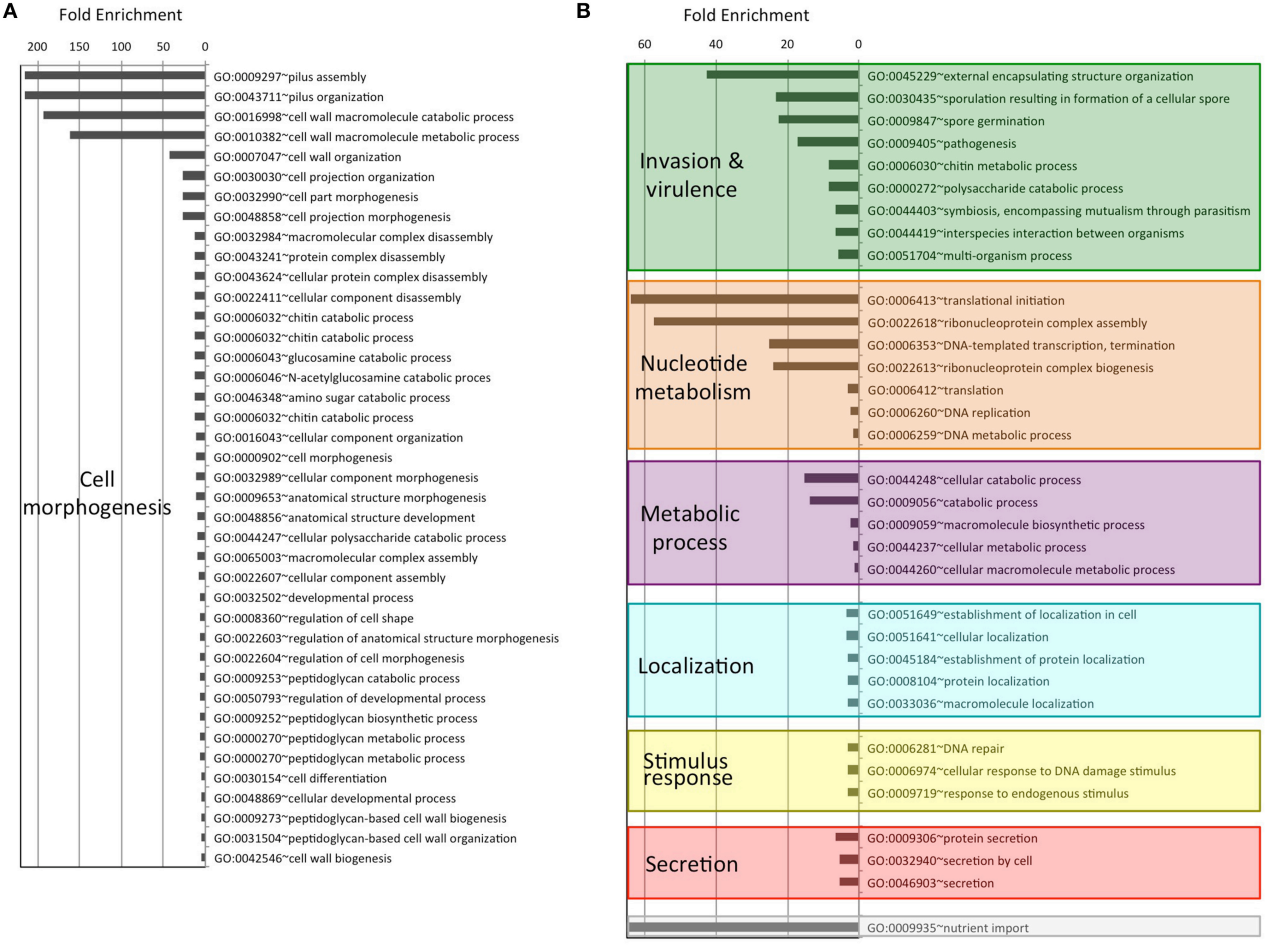
**Enrichment and clustering of bacteria PrD-containing proteins accordingly to their biological process GO terms**. The enrichment analysis was performed with GOStat against the goa_uniprot database. **(A)** Proteins with GO terms associated with cell morphogenesis. **(B)** Proteins with GO terms associated to other biological processes.

**Figure 2:**
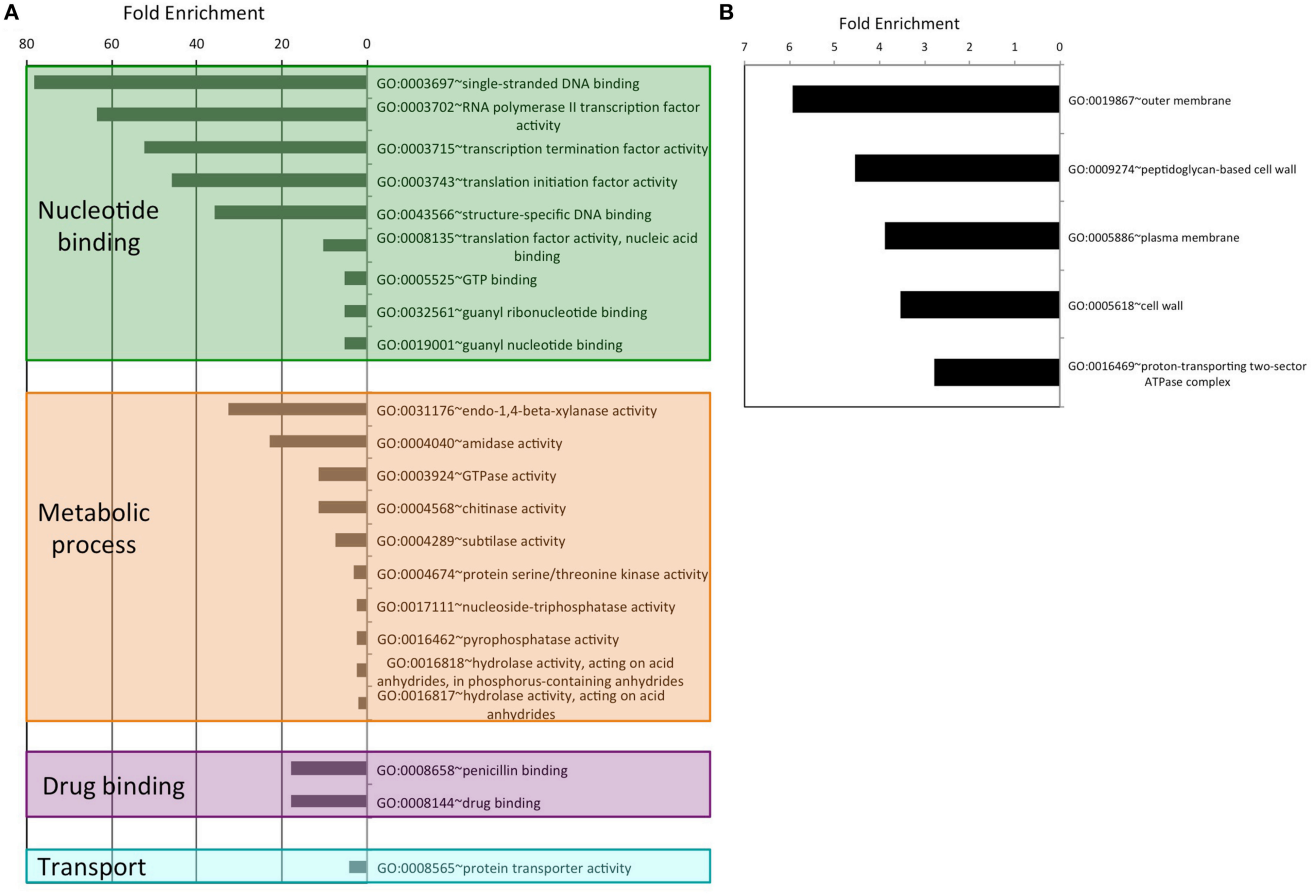
**Enrichment and clustering of bacteria PrD-containing proteins accordingly to their GO terms**. **(A)** Molecular function GO terms. **(B)** Cellular component GO terms.

As an attempt to understand the biological purpose of these PrDs we analyzed the Gene Ontology of the corresponding proteins. Additionally, to facilitate its interpretation we have grouped the enriched GO terms by similar cellular function or process. After this classification, the biggest cluster of GO terms collects Biological Processes involved in cell morphogenesis, such as cell projection or cell wall dynamics. This group contains 40 different terms, some of them with fold enrichments above 200 (pilus assembly; Figure 1A). We also found several enriched Biological Processes involved in secretion, nutrient import, invasion and virulence; all of them processes involved in interaction with the surrounding environment. Interestingly, in invasion and virulence we find processes associated to encapsulation, sporulation, and interaction with other organisms. Between the Biological Processes, the metabolic ones are particularly involved in the assembly of macromolecules such as polysaccharides and peptidoglycan (Figure 1B). The other three Biological Processes clusters are nucleotide metabolism, stimulus to response and localization, which are associated to cellular adaptation and the formation of contacts between molecules. When we analyse the Molecular Functions (Figure 2A), the GO terms enriched can be grouped as: nucleic acid binding, metabolic processes, drug binding, and transport. All of them activities associated with the formation of functional interactions. Additionally, the clusters of metabolic process and drug binding perform functions related to cell wall such as peptidoglycan synthesis or chitin production. Moreover, nucleic acid binding functions could be associated to mechanisms of cellular adaptation. The proteins in this cluster are strongly associated to two essential functions such as translation initiation and DNA templated transcription. Surprisingly, the GO terms of the Cell Component do not include any inside part of the cell, just terms associated to the external part: outer membrane, peptidoglycan based cell wall, plasma membrane, cell wall, and proton transport in flagella (Figure 2B; Namba, 2001). It is clear that many of the detected proteins, and specifically those involved in nucleotide binding, are located at the cytosol; however, because the large majority of bacterial proteins are categorized as cytosolic, this results in a poor enrichment factor for this compartment. Overall, the most remarkable characteristics of the bacteria proteins containing PrDs are their role in contact formation (e.g., macromolecular assembly), their relationship with the cell periphery and their involvement in nucleic acid mediated processes.

### Structural domains linked to bacteria PrD proteins

**Figure 3:**
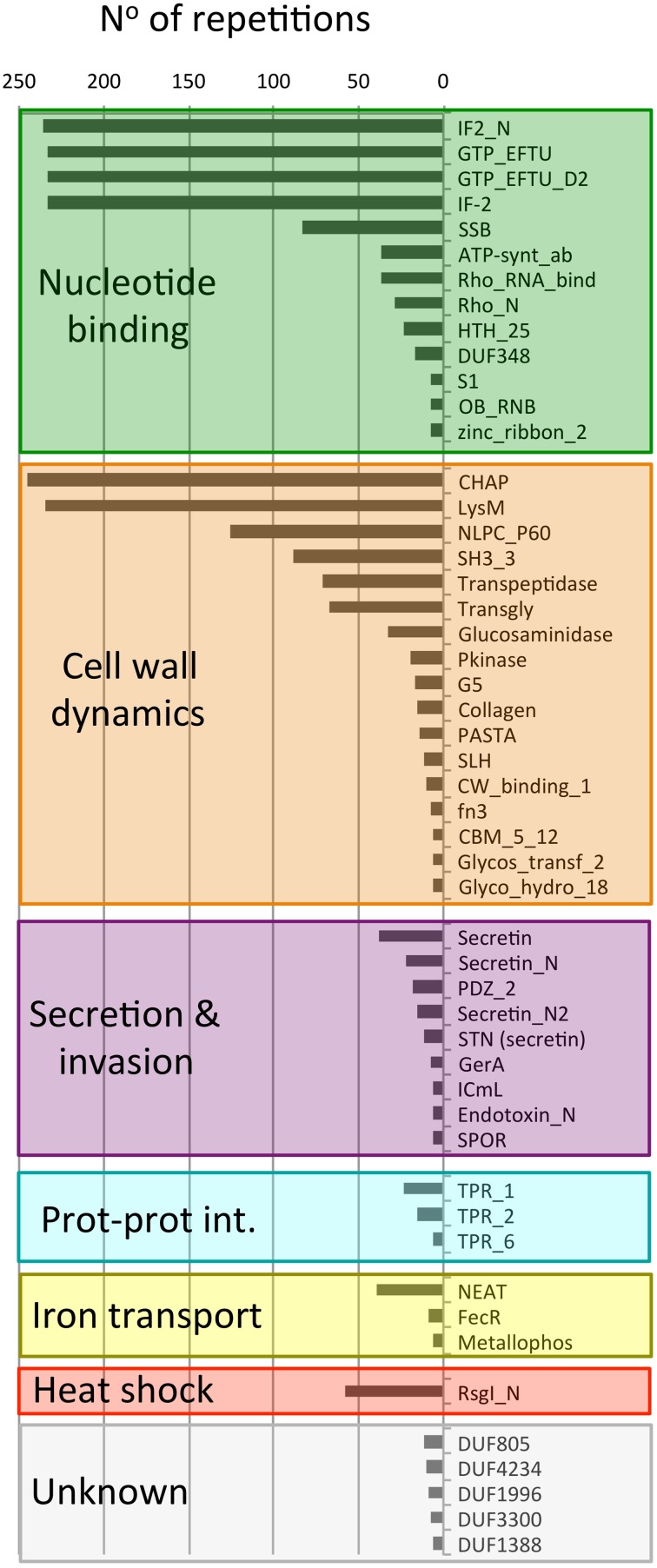
**Number of different Pfam domains found in PrD-containing proteins**. The domains are indicated by their Pfam ID. This plot only shows the domains with >5 repetitions in the dataset.

To learn more about the bacterial proteins that possess putative PrDs we examined their constituent functional domains (Finn et al., 2014; Figure 3). After clustering the Pfam domains we obtained eight functional groups: nucleotide binding, cell wall dynamics, invasion and virulence, protein-protein interaction, iron transport, heat-shock, and unknown.

The most abundant group of Pfam families is the one involved in nucleotide binding (1183 domains). There are included domains associated to translation such as GTP-binding elongation factors (GTP_EFTU), Rho termination factors (Rho_RNA_bind and Rho_N) and translation initiation factors (IF2 and IF2-N). Canonical nucleotide binding domains are also be found such as the single stranded binding protein (SSB), the single zinc ribbon domain (zinc_ribbon_2), the major structural motif helix-turn-helix (HTHth-25), and the S1 RNA binding domain. Finally, in this group we can also find an ATP synthase domain, associated with Rho termination factors (ATP-synt_ab), and the Ribonuclease B OB domain (Finn et al., 2014).

The second most abundant group of Pfam families is, once again, associated to cell wall dynamics (978 domains). This group clusters domains involved in cell wall metabolism (including biosynthesis and degradation) and proteins that bind the wall to build functional structures. For example, the lysine motif (Lysm) is involved in bacterial cell wall degradation and may also have peptidoglycan binding function (Bateman and Bycroft, 2000). The Glucosaminidase, Glycosyl transferase family 2 (Glycos_transf_2) and Transpeptidase are three domains associated with the biosynthesis of polysaccharides and peptidoglycan (Finn et al., 2014). We also found 67 proteins with a transglycosylase domain (Transgly) that catalyze the polymerization of murein glycan chains as well as 12 proteins with a SLH domain that is associated with the assembly of (glyco)proteins that coat the bacteria surface. The PASTA domain is involved in cell wall biosynthesis and can bind the beta-lactam rings enclosed in antibiotics. The most abundant domain from this group is the CHAP domain (245 proteins) with an amidase activity implicated in cell wall metabolism. Other domains also linked to cell wall are: the collagen domain (connective structures), the NlpC/P60 family (Anantharaman and Aravind, 2003; peptidases associated to lipoproteins), the G5 domain (adhesion), the fibronectin type III (fn3, adhesion), the cell wall binding motif 1 (CW_binding_1, a repeat similar to some clostridia toxins) and the carbohydrate-binding module (CBM_5_12, enriched in chitinases and associated to cellulose scaffolding). Additionally, the unknown domain DUF1388 has also been associated with surface lipoproteins.

The third group contains 130 proteins with domains associated to secretion and invasion. Here we have several domains associated to sporulation (SPOR) and spore germination (GerA). The secretin domains are involved in protein export via pore formation in a signal sequence-dependent manner (Van der Meeren et al., 2013; Tosi et al., 2014). The PDZ domains maintain together and organize signaling complexes located throughout the cellular membranes. Finally, the macrophage killing protein domain (ICmL) and the Endotoxin_N are domains involved in the formation of pores at the host cell membrane (Finn et al., 2014).

Between the PrD containing proteins we have also found three different tetratricopeptide repeat domains (46 repetitions), which scaffold protein-protein interactions and mediate the assembly of multi-protein complexes. In addition, we also obtained 54 domains linked to iron binding and transport (Metallophos, NEAT and FecR) and 58 proteins involved in heat shock response (Anti-sigma factor N-terminus), both types of domains aimed to interact with or to transduce signals coming from the cell external microenvironment.

Overall, the functional families of the PrD containing proteins (Figure 3) match very well with their GO classifications (Figures 1, 2) and confirm that these proteins are associated to the external part of the cell (e.g., cell wall) and interactions with other molecules (e.g., nucleotide binding).

### Structure composition of bacteria PrD containing proteins

**Figure 4:**
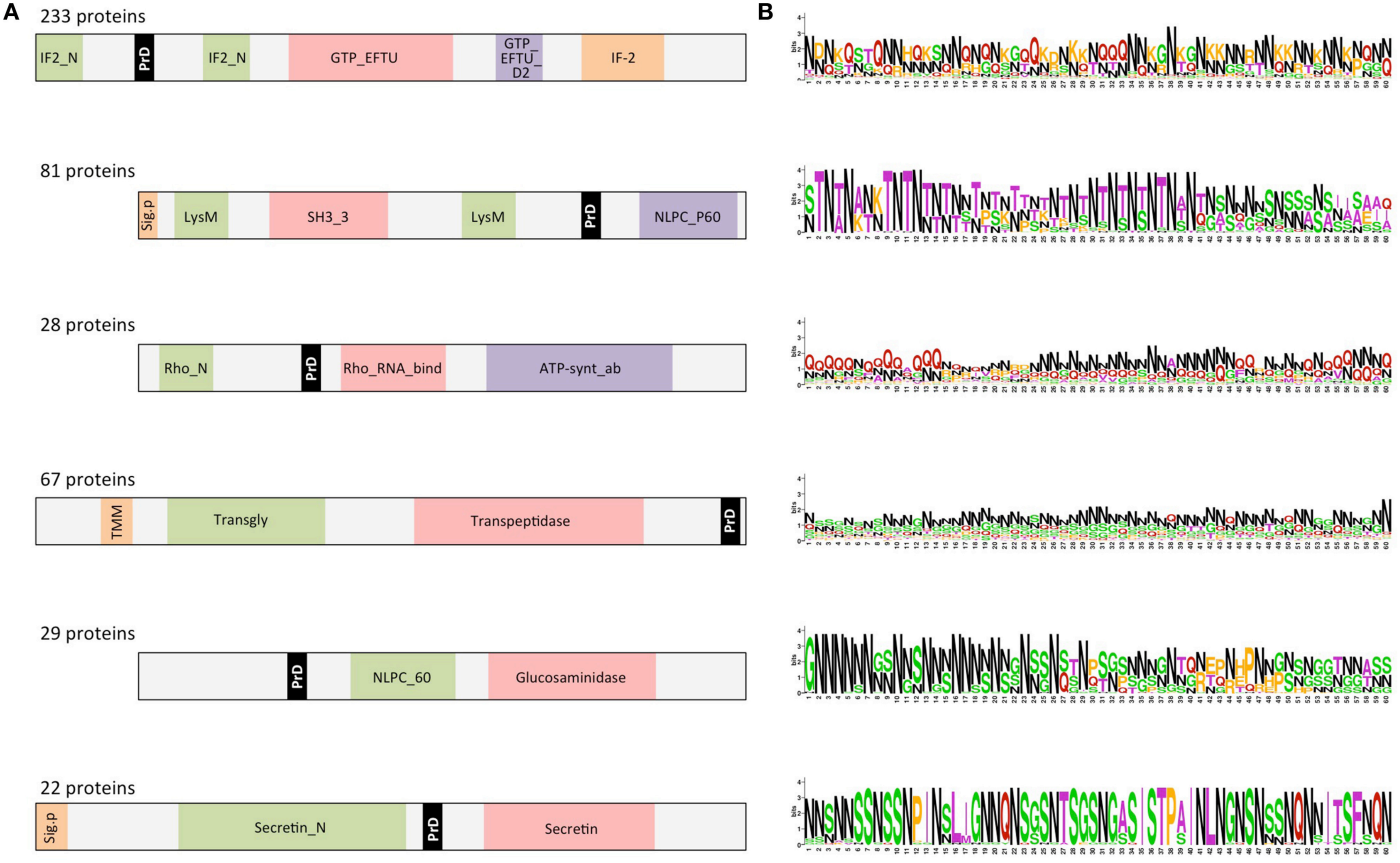
**PrD-containing proteins also contain multiple domains**. **(A)** Diagrams showing a consensus distribution and size of the most common domain combinations as collected in Pfam. The gray spaces indicate low complexity regions (coiled coil, disorder, etc). The domains are indicated by their Pfam ID. **(B)** PrD sequence conservation measured in bits. The symbol height reflects the relative frequency of the corresponding amino acid at that position. Color code: N in black; G in red; G, S, and Y in green; the other residues in purple (Crooks et al., 2004).

**Figure 5:**
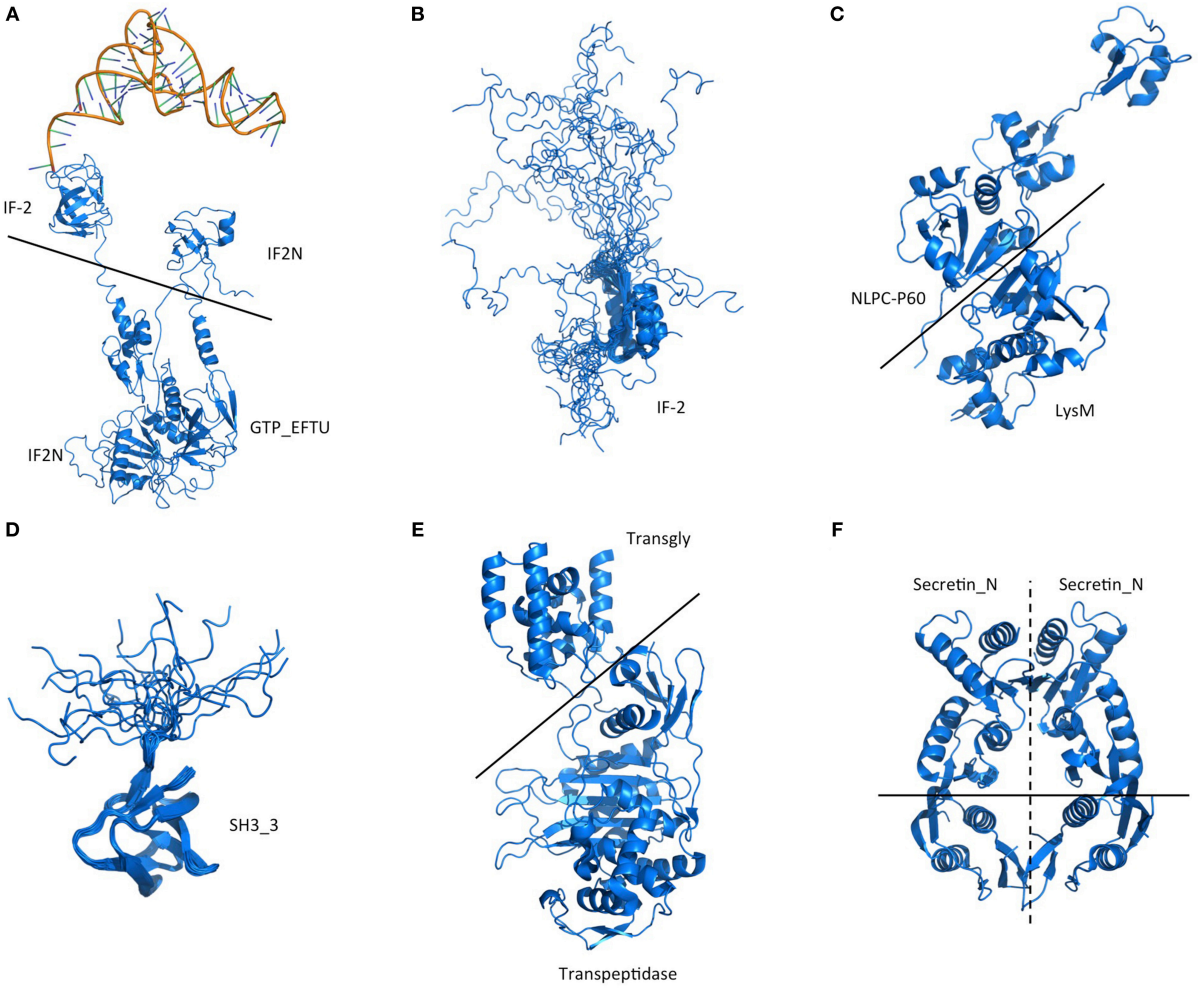
**Structure of the domains located in the PrD-containing proteins**. Representative structures of the domains and domain combinations enclosed in the PrD-containing proteins. The domains are indicated by their Pfam ID. **(A)** Example of quaternary complex where a multi-domain structure, composed by IF-2, IF2_N, and GTP_EFTU domains, interact with a tRNA. The image shows partial information from the PDB structure 1MJ1. Fitting the ternary complex of EF-TU/tRNA/GTP and ribosomal proteins into a 13 angstroms cryo-EM map of the *E. coli*'s 70S ribosome. **(B)** Example of IF-2 domain structure and the different states of the disordered region located in front of it. PDB structure 1z9b. Solution NMR structure of the C1-subdomain of *Bacillus stearothermophilus* translation initiation factor IF2 (fragment 515–635). **(C)** Example of multi-domain structure composed by a NlpC-P60 and a Lysm domains. PDB structure 4XCM. Crystal structure of the putative NlpC/P60 D,L endopeptidase from *Thermus thermophilus*. **(D)** Example of SH3_3 domain structure and the different states of the disordered region located after it. PDB structure 2KRS. Solution NMR structure of SH3 domain from CPF_0587 (fragment 415–479) from *Clostridium perfringens*. **(E)** Example of multi-domain structure composed by a transglycosylase and a transpeptidase domain. PDB structure 3ZG7 Crystal Structure of Penicillin-Binding Protein 4 from *Listeria monocytogenes* in the apo form. **(F)** Structure showing a homodimer constituted by Secretin_N domains. PDB structure 4E9J. Crystal structure of the N-terminal domain of the secretin XcpQ from *Pseudomonas aeruginosa*. Notice that at the multi-domain structures **(B,D)** the low complexity regions are abundant.

As expected, the detected PrDs are located inside low complexity regions (e.g., disordered, coiled coil, etc; Figures 4, 5). Moreover, these regions are abundant in the PrD containing proteins and connect different domains (Figure 4) and elements with secondary structure (Figure 5).

**Figure 6:**
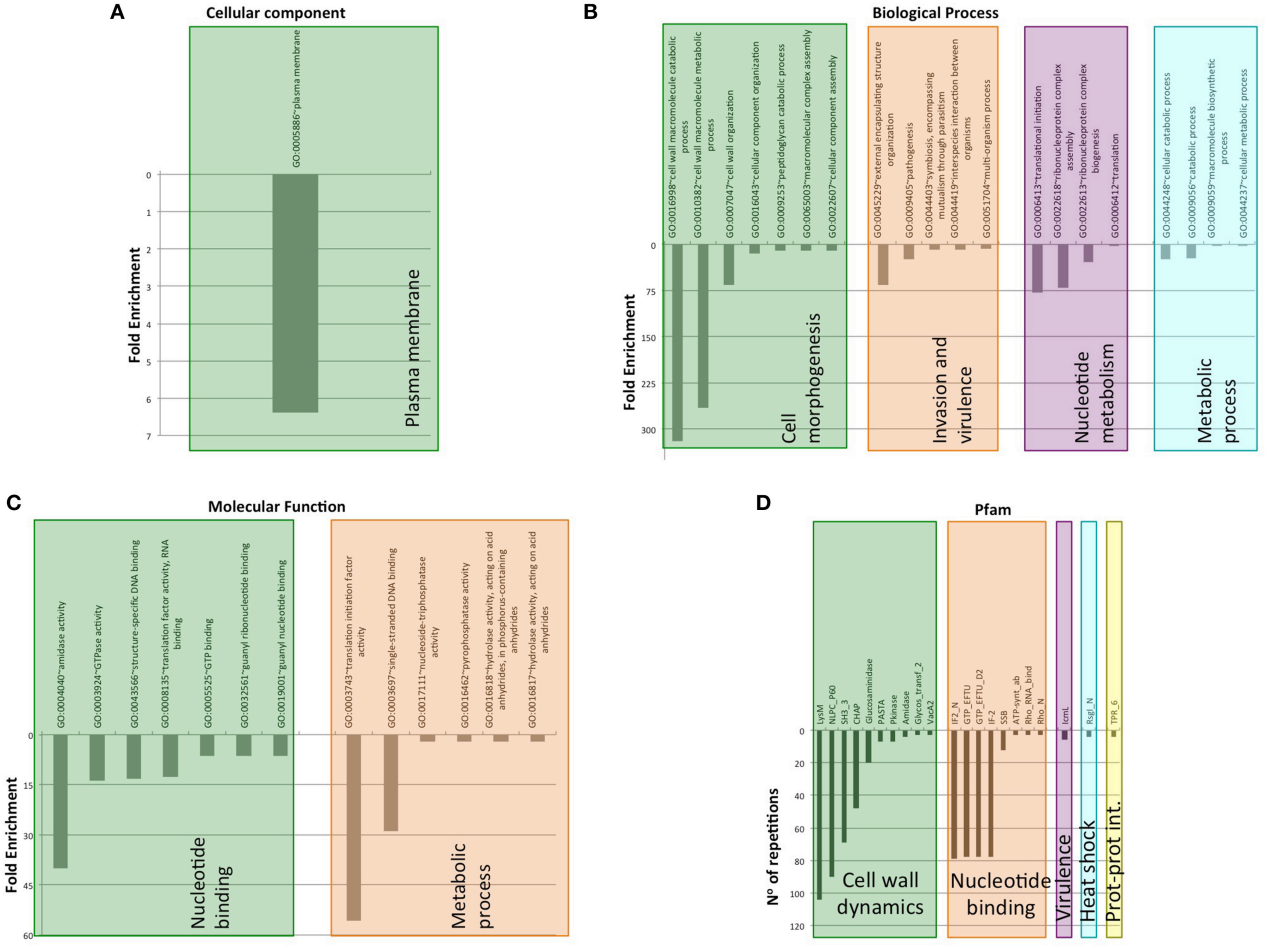
**Clustering of GO terms and Pfam domains associated to PrD-containing proteins in pathogen bacteria**. **(A)** Cellular component GO terms. **(B)** Biological Process GO terms. **(C)** Molecular Function GO terms. **(D)** Pfam domains associated.

From 2200 PrD containing proteins, 1514 have at least one defined Pfam domain (69%). Additionally, 612 of these sequences (40%) have more than one structural domain (Ekman et al., 2005). When we focus on the PrD containing proteins from pathogenic bacteria (Figure 6), we observe that they have a lower number of designated Pfam domains (just 301 proteins) suggesting they could be less structured proteins or, more likely, carry still unknown domains and functions. Despite this, the proteins from pathogenic bacteria with reported Pfam domains tend to contain more than one structural domain family (Table S2). The percentage of proteins with multiple domains appears to be higher in these proteomes (60%) than in the complete protein dataset (Ekman et al., 2005).

When the proteins have multiple structural domains, the PrD regions can be located either close to an end or between structures (Figure 4A). Interestingly, the amino acid composition of the PrD regions is similar between proteins sharing similar domain arrangement but different between proteins with distinct domains composition (Figure 4B). In agreement with the data reported for yeast prions, we observe that the detected regions are abundant in N (30%), Q (21%), S (11%), and G (11%).

The domain combinations tend to be functionally associated. For example, we found 233 protein sequences containing two GTP-binding elongation factor domains and two translation initiation factor domains that are related with nucleotide binding and translation (Figure 4). During protein synthesis the initiation factors (IF2) form a ternary complex with GTP and the initiator Met-tRNA (Wienk et al., 2005). This complex binds the ribosome to interact with the AUG-codon of the starting methionine, once the codon is found IF2 has to hydrolyze its GTP to be released (Figures 5A,B).

P60 domain is a cell-wall-associated peptidase domain essential for adherence and invasion in some Listeria species. In agreement with previous studies (Ponting et al., 1999; Anantharaman and Aravind, 2003), we observed the P60 domain associated with SH3 and LysM domains (Figures 4, 5C,D). It has been hypothesized that this team facilitates the domains interaction with peptides, carbohydrates and lipids from the bacterial cell wall and thus their functionality (Ponting et al., 1999; Anantharaman and Aravind, 2003).

Rho factor proteins tend to be accompanied with an RNA-binding domain and an ATP-hydrolysis domain (Figure 4). The Rho termination factor disengages newly transcribed RNA from its DNA template. Rho catalyzes the 3′ endpoint formation and the release of mRNA molecules from DNA templates (Skordalakes and Berger, 2003). The hydrolysis of ATP provides the energy required to get the RNA-DNA region and break the hybrid structure.

Another example of functional domain combination that contains PrDs are the penicillin-binding proteins. They are bifunctional proteins involved in the final stages of the peptidoglycan synthesis (Figures 4, 5E). At the N-terminus there is a transglycosylase domain involved in the formation of linear glycan strands. And at the C-terminus there is a transpeptidase domain involved in the cross-linking of peptide subunits and drug binding, which is also responsible of the penicillin-sensitivity (Macheboeuf et al., 2005; Sauvage et al., 2008; Contreras-Martel et al., 2011).

NLPC/P60 and Glucosaminidase are two cell wall endopeptidase domains, which emerged together and that we have found accompanied with a PrD (Figure 4). These two domains are commonly employed to cleave the septa connecting the daughter cells during cell separation (Anantharaman and Aravind, 2003; Ruggiero et al., 2010).

The secretins are another example of domain combination found in our set of PrD bacteria containing proteins (Figures 4, 5F). Particularly it is the most abundant combination of two domains (67 times) found in the PrD containing proteins. The secretin domains detected take part in protein secretion systems type II and III. They build multimeric pores to transport macromolecules either to the periplasm or to inject them into eukaryotic cells (Tosi et al., 2014). In general, secretin proteins consist of two domains: an N-terminal periplasmic domain responsible of the pore formation and a C-terminal domain responsible of the attachment to the outer membrane (Van der Meeren et al., 2013; Tosi et al., 2014). Interestingly, the PrD domain detected is located between these two secretin domains (Figure 4).

## Discussion

### Bacterial PrDs are associated to cellular adaptability

We observed that a significant fraction of the bacteria PrD containing proteins are located at the cell periphery and are involved in cell wall metabolism, especially peptidoglycan biogenesis. Peptidoglycan is the major component of bacterial cell walls; it is essential for growth, cell division, and maintenance of the cellular shape, enabling the bacteria to resist intracellular pressures of several atmospheres. In some particular cases, the proteins present in the peptidoglycan can be anchored to the biofilm amyloid network and, more interesting, assist its assembly. This is the case of the TapA protein from *B. subtilis*, which is present in the peptidoglycan, where it functions as an anchor point for TasA fibers. (Sauvage et al., 2008; Romero et al., 2011; Friedland, 2015). The formation of biofilms is a powerful strategy that protects a bacterial community from chemicals and antibiotics and facilitates the attachment to different surfaces even host cells. Interestingly, *S. aureus*, a biofilm forming pathogen, is the bacteria specie with the highest content in PrDs. In this organism we found PrD-containing proteins linked to cell wall, proteins involved in secretion and proteins associated to virulence. These data point to a possible relationship between the identified proteins and the biofilm formation. In fact, the *S. aureus* PrD-containing protein staphylococcal secretory antigen ssaA2 (Uniprot code Q2G2J2) is able to form amyloid fibrils *in vitro* (S.V. unpublished results). Thus, a more exhaustive analysis of these proteins might confirm their association to biofilms formation and their possible role as a drug targets.

The other processes enriched in the PrD containing proteins can also provide versatility and adaptability to different environments. For instance, the proteins involved in stimulus response and invasion and in virulence have a clear role in supporting the bacteria development under variable conditions. From inside the cell the nucleotide binding proteins can be involved in functions that support cell adjustment such as transcription and translation (i.e., change the expression levels) or DNA repair that can enhance cell survival in stress conditions. Interestingly, most of the novel prion-like proteins discovered recently in humans play a role in RNA/DNA binding (King et al., 2012). In bacteria, we also found proteins involved in cellular localization that can rearrange different compounds adapting the cell to new requirements. Overall, as previously proposed for yeast prions, bacterial prions might serve as bet-hedging devices for diversifying microbial phenotypes.

### Bacterial PrDs are associated to functional and interacting proteins

The 69% of PrDs containing proteins have defined Pfam domains and 40% of them carry multiple domains. Since domains come together to increase proteins functionality (Anantharaman and Aravind, 2003; Alberti et al., 2009), our data suggest that the proteins with PrDs tend to be functional. Moreover, in pathogenic bacteria PrD are associated to higher percentage of proteins with multiple domains, more than the average of the proteomes from this taxon (Ekman et al., 2005). This data suggests that, in pathogenic bacteria, PrD containing proteins might have a versatile character.

The detected PrDs are located in proteins rich in low complexity regions. These regions are important to provide the structural flexibility required to form interactions between proteins. This flexibility also allows the formation of reversible interactions, which are essential to build dynamic macromolecular assemblies. In fact, the GO terms associated to the PrDs detected by PrionScan comprise functions and processes linked to interaction and assembly. Many of these GO terms involve binding proteins, nucleotides or other cellular compounds. Human RNA/DNA binding proteins use their PRDs to attain functional macromolecular assemblies that regulate transcription and translation. In many cases these functions are exerted in the so called ribonucleoprotein granules (Malinovska et al., 2013). Many of the proteins containing DNA/RNA binding domains identified in the present also work by forming large complexes and indeed are implied in ribonucleoprotein complex biogenesis and assembly suggesting that this property can be conserved across species. In addition, the association to cell wall dynamics suggests that certain proteins can be implied in the assembly and disassembly of peptidoglycans and polysaccharides. Overall, our data supports that, as previously suggested for eukaryotic PrDs, bacteria PrDs could play an important role in the arrangement of macromolecular structures (Malinovska et al., 2013).

### Prions in other proteomes

*Saccharomyces cerevisiae* is the organism from which more information about its prion proteins has been so far collected (Alberti et al., 2009; Malinovska et al., 2013). These works showed for the first time that proteins could be employed for amazing functions such as epigenetic elements essential to adapt the cellular metabolism and increase the cell survival in front of environmental changes (Alberti et al., 2009; Newby and Lindquist, 2013). In *S. cerevisiae* the prion proteins are associated to functions that involve the formation of contacts such as RNA-binding, membrane-interacting, DNA binding and protein interaction domains (Malinovska et al., 2013). These proteins are located at the cytoskeleton, nucleus, ribonucleoprotein complexes, and chromatin. Comparing *S. cerevisiae* with other eukaryotic proteomes shows PrD-containing proteins with similar function and location. For example, in human and fruit fly these proteins are also involved in transcription, chromatin remodeling, ribonucleoprotein complex formation, and cytoskeleton (Malinovska et al., 2013). In animals, PrDs tend to be involved in the regulation of central biological processes and organism development, which in vertebrates includes the development of the neural crest. Hence, many human PrD are found in RNA-binding proteins, which deregulation has previously been associated with several neurodegenerative diseases (King et al., 2012).

Eukaryote PrD-containing proteins show less functional diversity than bacteria. In fact, here we have collected all the enriched eukaryote functions (i.e., transcription, RNA binding, and DNA binding) in just one cluster (nucleotide binding). Despite this difference, it appears that, independently of the considered taxon, PrD-containing proteins appear to be involved in a similar regulatory purpose: adapting the cell to a variable environment. This purpose is basically achieved through the control of the expression in eukaryotes, but in prokaryotes this is also reached by interacting with the environment, since microorganisms face the constant challenge of fluctuating conditions in their natural environments. These strategies may have facilitated the invasion of new environments (e.g., water, air) and the coexistence or exploitation of diverse life forms (e.g., host cells).

### Bacteria PrDs and human diseases

Our life is closely linked to bacteria, either through a parasitic or symbiotic relationship. On one hand, human microbiota is required to assist many processes and ensure a healthy body. On the other hand, many common pathogenic bacteria are acquiring antibiotic resistance in all regions of the world (e.g., urinary tract infections, pneumonia, bloodstream infections; WorldHealthOrganisation, WHO). These bacteria cause many hospital-acquired infections, such as the methicillin-resistant *S. aureus*, with an associated high mortality rate (Contreras-Martel et al., 2011; WorldHealthOrganisation, WHO).

To the already intricate scenario where bacteria and host interact, the risk of their amyloid proteins concurring and altering their conformational states adds an extra level of complexity (Otzen and Nielsen, 2008). Additionally, the long periods that bacteria stay in the body, due to chronic infection or microbiota coexistence, enhances the chances of this event. In fact, recent studies have demonstrated that bacterial amyloids can initiate the formation of amyloid aggregates upon interaction with host proteins (Otzen and Nielsen, 2008; Zhou et al., 2012; Hufnagel et al., 2013; Hill and Lukiw, 2015). Moreover, it has been reported that the injection of bacteria amyloids in mice causes the development of amyloidosis (Lundmark et al., 2005). Overall, these data reminds the conformational template process associated to prion transmission and suggest that bacterial infection could be linked to neurodegenerative diseases (Friedland, 2015).

### General conclusions

Despite PrD-containing proteins seem to be ubiquitous (Espinosa Angarica et al., 2013; Malinovska et al., 2013) they play distinct functional roles in different species. In this background, the mechanisms underlying host-bacteria relationship are just starting to be elucidated and, as a result, also the interplay between their amyloid proteins (Zhou et al., 2012; Schwartz and Boles, 2013; Seviour et al., 2015). The studies on bacteria amyloids are showing us that amyloid aggregates can be exploited to execute wide range of amazing functions (Blanco et al., 2012; DePas and Chapman, 2012; Gsponer and Babu, 2012; Zhou et al., 2012; Schwartz and Boles, 2013; Evans et al., 2015; Seviour et al., 2015; Taylor and Matthews, 2015). Because the formation of amyloids comes at expenses of the formation of transient toxic species cells tightly control the assembly of these macromolecular structures and how they can interact with proteins from other species (Zhou et al., 2012; Schwartz and Boles, 2013; Evans et al., 2015; Taylor and Matthews, 2015). Most of the bacterial amyloids described so far play a structural role and work extracellularly. Indeed, some of the PrD containing proteins with potential amyloidogenic properties could be linked to biofilms, structures that favor chronic human infections and, consequently, increase the chances of a potential bacterial prion to alter the conformation of host proteins. However, despite their *in vitro* amyloid potential and *in vivo* prionic behavior should be validated, the data in the present work suggest that, as it happens in yeast and humans, also in bacteria amyloid-like assemblies might play a regulatory role, since some of the detected candidates are linked to fundamental cellular functions such as transcription, translation or DNA repair. Intriguingly, linking the fact that we found at the same time association with extracellular environment and nucleic acid binding function, it has been reported recently that extracellular DNA is bound tightly by bacterial amyloid fibrils during biofilm formation and that amyloid/DNA composites are immune stimulators when injected into mice, leading to autoimmunity (Gallo et al., 2015; Spaulding et al., 2015). Overall it becomes clear that a more exhaustive analysis of the putative bacterial prion proteins identified here is required in order to attain a better understand of their functional role and their relationship with human diseases. This data could help to identify new drug targets and develop new therapies.

### Conflict of interest statement

The authors declare that the research was conducted in the absence of any commercial or financial relationships that could be construed as a potential conflict of interest.

## Footnotes

^1^http://www.who.int/mediacentre/factsheets/fs194/en/.
